# An Evaluation of the Hematological Markers of Systemic Inflammation and Oxidative Stress in Vitiligo: A Case-Control Study

**DOI:** 10.7759/cureus.56034

**Published:** 2024-03-12

**Authors:** Berkay Temel, Ozge Orenay, Nermin Karaosmanoglu

**Affiliations:** 1 Dermatology, Ankara Training and Research Hospital, Ministry of Health, Ankara, TUR; 2 Dermatology, Ankara Training and Research Hospital, Ankara, TUR; 3 Dermatology, Ankara Education and Research Hospital, Health Sciences University, Ankara, TUR

**Keywords:** stress, oxidative, monocyte, neutrophil, vitiligo

## Abstract

Introduction

Melanocyte dysfunction in vitiligo is considered to be due to genetics, inflammation, and autoimmunity. Research has shown that oxidative stress plays a significant role in triggering these conditions. Currently, there are several markers indicating hematological inflammation and oxidative stress. This study aimed to investigate the status of inflammation and oxidative stress markers in vitiligo.

Methods

This study included patients with vitiligo and age-gender-matched healthy controls. C-reactive protein (CRP), neutrophil-to-lymphocyte ratio (NLR), monocyte-to-lymphocyte ratio (MLR), platelet-to-lymphocyte ratio (PLR), and monocyte-to-high-density lipoprotein ratio (MHR) and extent of vitiligo were calculated and compared.

Results

The study included 138 participants (69 vitiligo and 69 controls). The mean was 41.46 years with a female predominance (55.1%). The patient group demonstrated higher levels of platelets, neutrophils, CRP, NLR, MLR, PLR, and HDL and lower levels of lymphocytes and HDL compared to the control group (p>0,05). The only significantly different value between the groups was MHR (p=0.03). The generalized vitiligo group demonstrated higher levels of platelets, neutrophils, monocytes, CRP, NLR, PLR, and MLR, and lower levels of lymphocytes and HDL compared to the localized group. The only significantly different values between the groups were MHR and MLR (p=0.02, p=0.03).

Conclusion

This study found that MHR and CRP values were higher in vitiligo patients. Additionally, MHR and MLR values were higher in patients with generalized vitiligo. These results suggest that MHR is a reliable indicator marker for systemic inflammation and oxidative stress in vitiligo.

## Introduction

Vitiligo is an autoimmune skin disease, characterized by white patches due to melanocyte loss. Vitiligo is the most common depigmented skin disease with a prevalence of 0.5-2% worldwide. The disease can be seen in all ethnic groups and skin types [1,2]. There are two main clinical types of vitiligo: segmental and nonsegmental vitiligo. Segmental vitiligo typically occurs in childhood and affects only one area of the body. Nonsegmental vitiligo is classified into acrofacial, mucosal, generalized, universal, mixed, and rare variants according to the area and extent of involvement [3].

Melanocyte dysfunction in vitiligo is considered to be due to genetics, inflammation, and autoimmunity. The cause of melanocyte dysfunction in vitiligo is attributed to inflammation and autoimmunity. Research has shown that oxidative stress plays a significant role in triggering these conditions. O2-, -OH, and H2O2 are the main types of reactive oxygen species (ROS). ROS are produced as a result of cellular metabolic processes or exogenous exposure. Melanin synthesis in cells is another source of ROS. Antioxidant systems typically destroy ROS formed inside the cell. However, excessive ROS production beyond the capacity of antioxidant systems can cause tissue and cell damage, leading to inflammation [4-6]. Inflammation is an adaptive response to potential stimuli that could harm the host. It is a four-step process consisting of trigger, sensor mechanism, signal generation, and effector response, all of which can be affected by ROS [7].

ROS overproduction in melanocytes can lead to the formation of autoantigens, which may trigger adaptive and cellular immunity [3]. Dendritic cells and macrophages are activated by autoantigens, resulting in T-cell activation. It is known that the activation of the Th1 response of helper and cytotoxic T cells increases the release of tumor necrosis factor (TNF)-α and interferon (IFN)-γ [8]. Activated macrophages are a significant source of ROS to eliminate harmful pathogens [7]. However, the overproduction of ROS by macrophages may be one of the causes of melanocyte injury.

Antioxidants are classified as enzymatic or non-enzymatic based on their structure. The primary enzymatic antioxidants include superoxide dismutase, catalase, and glutathione peroxidase. The primary non-enzymatic antioxidants include vitamin A, vitamin C, vitamin E, uric acid, and beta-carotene [9]. Besides these antioxidants, high-density lipoprotein (HDL) is also known to have antioxidative properties. Its primary function is to prevent the development of atherosclerosis by protecting LDL from oxidation [10].

Currently, new biomarkers have been developed by comparing various hematological and biochemical laboratory values. These markers have been studied for their ability to predict inflammation burden, disease prognosis, or treatment follow-up [11]. The major biomarkers are neutrophil-to-lymphocyte ratio (NLR), monocyte-to-lymphocyte ratio (MLR), and platelet-to-lymphocyte ratio (PLR). Recently, it has been suggested that the monocyte to HDL ratio (MHR) is a significant marker for inflammation and oxidative stress, particularly in the prognosis of cardiovascular diseases [12]. This study aimed to investigate the relationship between hematological inflammatory and oxidative stress markers and vitiligo.

## Materials and methods

Patient selection, study plan, and evaluation criteria

This study was approved by the local ethics committee and was in accordance with the Declaration of Helsinki (93471371-514.10-E/20488). The study included patients with vitiligo and age-gender-matched healthy controls between August 2015 and September 2020. The study excluded patients with additional acute or chronic infectious diseases, hematological diseases, inflammatory diseases, obesity, and smoking. Study variables, such as age, gender, percentage of the body surface area (BSA) involvement, neutrophil count, lymphocyte count, monocyte count, platelet count, HDL value, and C-reactive protein (CRP) value, were recorded from the patient's electronic medical records. The calculation of the percentage of body surface area (BSA) affected by vitiligo was performed using the rule of nines (palmar hand accounts for approximately 1%). NLR, MLR, PLR, and MHR were calculated by proportioning each other. The study variables were compared between patients with vitiligo and controls.

Statistical analysis

The research data were analyzed using the SPSS 22.0 package (IBM Corp., Armonk, NY, US). The normal distribution compatibility of the variables was analyzed using Kolmogorov-Smirnov/Shapiro-Wilk tests. Categorical variables were presented as numbers and percentages in the descriptive statistics section. The continuous variables were presented as mean ± standard deviation for normally distributed data and median (minimum-maximum value) for non-normally distributed data. The difference in categorical data between the two independent groups was evaluated by the chi-square test. Pearson correlation analysis was used to measure the linear correlation between the two datasets. The statistical significance value of p<0.05 was accepted.

## Results

Information about patients and controls

The study included 138 participants, 69 of them with vitiligo and 69 of them controls. The mean age of the patients was 41.46 years and there was a female predominance (55.1%, n=38). There was no statistically significant difference between the patient and control groups in terms of age and gender (Table 1).

**Table 1:** The comparison of patient and control groups in terms of study variables *: T-test, **: chi-square test, ***: Mann-Whitney U test SD: standard deviation, HDL: high-density lipoprotein, CRP: C-reactive protein, MHR: monocyte-to-lymphocyte ratio, NLR: neutrophil-to-lymphocyte ratio, PLR: platelet-to-lymphocyte ratio, MLR: monocyte-to-lymphocyte ratio

	Patient (n=69)	Control (n=69)	p-value
Age, mean±SD	41.46±15.35	40.4±10.9	0.82*
Gender, n (%): Female Male	38 (55.1) 31 (44.9)	38 (55.1) 31 (44.9)	0.9**
Platelet, 10^9^/L	266.89	251.05	0.17*
Neutrophil, 10^9^/L	4.09	3.61	0.06*
Lymphocyte, 10^9^/L	2.22	2.30	0.98*
Monocyte, 10^9^/L	0.55	0.50	0.09*
HDL, mg/dl	48.8	56.6	0.01***
CRP, mg/l	3.19	1.72	0.04***
MHR	0.012	0.007	0.04*
NLR	1.98	1.73	0.08***
PLR	131.2	121.5	0.49***
MLR	0.26	0.23	0.23***

Comparison of groups in terms of hematologic parameters and markers

The patient group demonstrated higher levels of platelets, neutrophils, and CRP, and lower levels of lymphocytes and HDL compared to the control group. The only significantly different value between the groups was CRP (p=0.04) (Table 1). The patient group demonstrated significantly higher NLR, MLR, PLR, and MHR values as compared to the control group. The only significantly different value between the groups was MHR (p=0.03) (Table 1).

Comparison of vitiligo groups in terms of hematologic parameters and markers

The study involved 36 patients with localized vitiligo and 33 patients with generalized vitiligo. The age and gender of the two groups did not differ significantly. The generalized group demonstrated higher levels of platelets, neutrophils, monocytes, CRP, NLR, PLR, and MLR, and lower levels of lymphocytes and HDL compared to the localized group. The only significantly different values between the groups were MHR and MLR (p=0.02, p=0.03) (Table 2).

**Table 2:** Comparison of localized and generalized vitiligo groups in terms of study variables *: T-test, **: chi-square test, ***: Mann-Whitney U test SD: standard deviation, HDL: high-density lipoprotein, CRP: C-reactive protein, MHR: monocyte-to-lymphocyte ratio, NLR: neutrophil-to-lymphocyte ratio, PLR: platelet-to-lymphocyte ratio, MLR: monocyte-to-lymphocyte ratio

	Localized (n=36)	Generalized (n=33)	p-value
Age, mean±SD	40.47	42.54	0.57*
Gender, n(%): Female, Male	20 (55.6), 16 (44.4)	18 (54.5) 15 (45.5)	0.93**
Platelet, 10^9^/L	255.1	279.7	0.11*
Neutrophil, 10^9^/L	4	4.2	0.9*
Lymphocyte, 10^9^/L	2.25	2.2	0.26*
Monocyte, 10^9^/L	0.52	0.59	0.07*
HDL, mg/dl	50.7	46.8	0.13***
CRP, mg/l	1.75	1.98	0.63***
MHR	0.08	0.16	0.02*
NLR	1.96	2.03	0.61***
PLR	120.94	142.52	0.11***
MLR	0.24	0.29	0.03***

Correlation analysis between hematologic parameters and markers

The correlation analysis of hematologic parameters and markers is shown in Table 3.

**Table 3:** The correlation analysis of hematologic parameters and markers Pearson correlation analysis, (-) refers to a negative correlation HDL: high-density lipoprotein, CRP: C-reactive protein, MHR: monocyte-to-lymphocyte ratio, NLR: neutrophil-to-lymphocyte ratio, PLR: platelet-to-lymphocyte ratio, MLR: monocyte-to-lymphocyte ratio

		Platelet	Neutrophil	Lymphocyte	Monocyte	HDL	CRP	MHDL	NLR	PLR	MLR
Platelet	Pearson Correlation	1	0.298^*^	-0.027	0.192	0.041	0.197	0.081	0.267^*^	0.670^**^	0.183
	p-value		0.013	0.826	0.114	0.736	0.124	0.507	0.027	0.000	0.132
Neutrophil	Pearson Correlation	0.298^*^	1	0.088	0.377^**^	-0.171	-0.059	0.337^**^	0.673^**^	0.086	0.191
	p-value	0.013		0.470	0.001	0.159	0.647	0.005	0.000	0.481	0.115
Lymphocyte	Pearson Correlation	-0.027	0.088	1	0.227	-0.240^*^	0.192	0.322^**^	-0.576^**^	-0.665^**^	-0.521^**^
	p-value	0.826	0.470		0.061	0.047	0.135	0.007	0.000	0.000	0.000
Monocyte	Pearson Correlation	0.192	0.377^**^	0.227	1	-0.153	-0.210	0.813^**^	0.159	-0.019	0.608^**^
	p-value	0.114	0.001	0.061		0.209	0.101	0.000	0.191	0.876	0.000
HDL	Pearson Correlation	0.041	-0.171	-0.240^*^	-0.153	1	0.012	-0.649^**^	0.021	0.159	0.060
	p-value	0.736	0.159	0.047	0.209		0.927	0.000	0.864	0.191	0.623
CRP	Pearson Correlation	0.197	-0.059	0.192	-0.210	0.012	1	-0.194	-0.137	0.022	-0.269^*^
	p-value	0.124	0.647	0.135	0.101	0.927		0.131	0.289	0.867	0.034
MHR	Pearson Correlation	0.081	0.337^**^	0.322^**^	0.813^**^	-0.649^**^	-0.194	1	0.057	-0.143	0.385^**^
	p-value	0.507	0.005	0.007	0.000	0.000	0.131		0.640	0.240	0.001
NLR	Pearson Correlation	0.267^*^	0.673^**^	-0.576^**^	0.159	0.021	-0.137	0.057	1	0.615^**^	0.624^**^
	p-value	0.027	0.000	0.000	0.191	0.864	0.289	0.640		0.000	0.000
PLR	Pearson Correlation	0.670^**^	0.086	-0.665^**^	-0.019	0.159	0.022	-0.143	0.615^**^	1	0.572^**^
	p-value	0.000	0.481	0.000	0.876	0.191	0.867	0.240	0.000		0.000
MLR	Pearson Correlation	0.183	0.191	-0.521^**^	0.608^**^	0.060	-0.269^*^	0.385^**^	0.624^**^	0.572^**^	1
	p-value	0.132	0.115	0.000	0.000	0.623	0.034	0.001	0.000	0.000	

## Discussion

Vitiligo is a chronic, relapsing skin disease with static and dynamic phases. The management of vitiligo is challenging for both patients and physicians. The chronic and relapsing nature of the disease makes it difficult to provide appropriate and sustainable treatment options. One of the difficulties in the treatment process is that clinical findings alone are insufficient to predict treatment response and prognosis. It is currently known that the etiopathogenesis of vitiligo is based on oxidative stress, inflammation, and autoimmunity [13]. Recent studies have identified several inflammatory and oxidative stress markers derived from hematological laboratory values [14]. This study investigated the ability of these new markers to indicate systemic inflammation and oxidative stress in vitiligo.

C-reactive protein (CRP) is a marker for systemic inflammation. Interleukin-1 (IL-1), interleukin-6 (IL-6), and tumor necrosis factor-alpha (TNF-α) mediate the modulation of the hepatic synthesis of acute-phase reactants, including CRP [15]. Namazi et al., Aryanian et al., Ghaderi et al., and Solak et al. investigated the role of CRP in vitiligo. The studies consistently found that CRP levels were elevated in vitiligo patients compared to the control group [16-19]. Furthermore, Ghaderi et al. and Solak et al. reported that CRP levels were significantly higher in patients with generalized vitiligo compared to those with localized vitiligo [18,19]. Our study also found that CRP levels were significantly higher in vitiligo patients compared to controls. However, no significant difference was detected between vitiligo severity groups in terms of CRP levels, although it tended to be higher in the generalized group. The variation in results could be attributed to differences in the number of patients and their characteristics across the studies. It is important to note that CRP levels can be affected by various factors such as smoking, high BMI, infections, inflammation, and cancer. Our study excluded these factors to ensure accurate results. Unlike previous studies, we do not consider CRP to be a reliable marker for indicating the severity of vitiligo or monitoring treatment response due to the presence of lots of confounding factors.

NLR is an inflammatory hematological marker that is associated with cardiovascular morbidity and mortality [20]. Solak et al., Mascarenhas et al., and Demirbaş et al. investigated the role of NLR in vitiligo [14,19,21]. Solak et al. found that the NLR level was significantly higher in generalized vitiligo compared to the control and localized vitiligo groups. However, there was no significant difference between the localized vitiligo and control groups. Furthermore, Solak et al. suggested that an elevated NLR may indicate an increased cardiovascular risk in vitiligo [19]. In contrast to Solak et al., both Mascarenhes et al. and Demirbaş et al. did not find a significant increase in NLR levels in vitiligo patients compared to the control group [14,21]. However, Demirbaş et al. did find a positive correlation between vitiligo severity and NLR [14]. Our study also found higher NLR values in vitiligo patients compared to controls, with even higher values in those with generalized vitiligo compared to localized vitiligo. However, the difference between the two groups was not statistically significant. The variation in results could be attributed to differences in the number of patients and their characteristics across the studies. At this stage, NLR does not seem to be a reliable marker for predicting treatment response and prognosis in vitiligo. Further researches are required to investigate the role of NLR in vitiligo through large-scale, multicenter studies.

MLR and PLR are currently considered hematological markers for indicating inflammation in diseases [22]. Demirbaş et al. investigated the role of MLR and PLR in vitiligo and found that it was statistically significantly higher in vitiligo patients as compared to the control group. They also found a positive correlation between the severity of vitiligo and MLR. However, our study found no statistically significant difference in MLR and PLR between patients with vitiligo and controls. However, only the MLR value was significantly higher in patients with generalized vitiligo. The variation in results could be attributed to differences in the number of patients and their characteristics across the studies. At this stage, MLR and PLR do not seem to be a reliable marker for predicting the treatment response and prognosis of vitiligo. However, further researches are required to investigate the role of MLR in vitiligo through large-scale, multicenter studies.

MHR is considered an indicator of atherosclerosis, systemic inflammation, and oxidative stress [23]. Demirbaş et al. investigated the role of MHR in vitiligo and found that it was significantly higher in patients with vitiligo compared to controls. They also reported a positive correlation between MHR and vitiligo severity [14]. Our study also found similar results, indicating that MHR could be a reliable marker for monitoring treatment response and determining prognosis. On the other hand, the relationship between vitiligo and cardiovascular disease is not clear in the literature and contradictory results have been obtained. A systematic review of seven studies found that six of them identified vitiligo as a risk factor for cardiovascular diseases. These studies have found an association between vitiligo and cardiovascular disease risk factors such as hypertension, dyslipidemia, and carotid artery media-intima thickness [24,25]. The literature also reports that MHR is an indicator of cardiovascular disease risk and is associated with atherosclerosis [23]. Therefore, high MHR in vitiligo patients may indicate an increased risk of cardiovascular disease. However, further researches are required to investigate the role of MLR in vitiligo through large-scale, multicenter studies.

The limitation of this study was that is a small-sized, single-center retrospective study.

## Conclusions

This study found that MHR and CRP values were higher in vitiligo patients as compared to controls. Additionally, MHR and MLR values were higher in patients with generalized vitiligo compared to localized vitiligo. These results suggest that MHR is a reliable indicator marker for systemic inflammation and oxidative stress in vitiligo. Furthermore, high MHR may be a useful marker for indicating the risk of cardiovascular diseases in vitiligo. There is a need for multi-participant multicenter studies to examine the role of hematological markers associated with systemic inflammation and oxidative stress in predicting the presence of vitiligo-related comorbidities, vitiligo treatment response, and prognosis.
